# Increased Prolactin Levels Are Associated with Impaired Processing Speed in Subjects with Early Psychosis

**DOI:** 10.1371/journal.pone.0089428

**Published:** 2014-02-24

**Authors:** Itziar Montalvo, Alfonso Gutiérrez-Zotes, Marta Creus, Rosa Monseny, Laura Ortega, Joan Franch, Stephen M. Lawrie, Rebecca M. Reynolds, Elisabet Vilella, Javier Labad

**Affiliations:** 1 Early Psychosis Program and Research Department, Hospital Universitari Institut Pere Mata, IISPV, Universitat Rovira i Virgili, CIBERSAM, Reus, Spain; 2 Division of Psychiatry, Kennedy Tower, Royal Edinburgh Hospital, University of Edinburgh, Edinburgh, United Kingdom; 3 Endocrinology Unit, University/BHF Centre for Cardiovascular Sciences, Queen’s Medical Research Institute, University of Edinburgh, Edinburgh, United Kingdom; University of Pécs Medical School, Hungary

## Abstract

Hyperprolactinaemia, a common side effect of some antipsychotic drugs, is also present in drug-naïve psychotic patients and subjects at risk for psychosis. Recent studies in non-psychiatric populations suggest that increased prolactin may have negative effects on cognition. The aim of our study was to explore whether high plasma prolactin levels are associated with poorer cognitive functioning in subjects with early psychoses. We studied 107 participants: 29 healthy subjects and 78 subjects with an early psychosis (55 psychotic disorders with <3 years of illness, 23 high-risk subjects). Cognitive assessment was performed with the MATRICS Cognitive Consensus Cognitive Battery, and prolactin levels were determined as well as total cortisol levels in plasma. Psychopathological status was assessed and the use of psychopharmacological treatments (antipsychotics, antidepressants, benzodiazepines) recorded. Prolactin levels were negatively associated with cognitive performance in processing speed, in patients with a psychotic disorder and high-risk subjects. In the latter group, increased prolactin levels were also associated with impaired reasoning and problem solving and poorer general cognition. In a multiple linear regression analysis conducted in both high-risk and psychotic patients, controlling for potential confounders, prolactin and benzodiazepines were independently related to poorer cognitive performance in the speed of processing domain. A mediation analysis showed that both prolactin and benzodiazepine treatment act as mediators of the relationship between risperidone/paliperidone treatment and speed of processing. These results suggest that increased prolactin levels are associated with impaired processing speed in early psychosis. If these results are confirmed in future studies, strategies targeting reduction of prolactin levels may improve cognition in this population.

## Introduction

Hyperprolactinaemia is a common condition in subjects with a psychotic disorder. As dopamine is the main prolactin inhibiting factor, hyperprolactinaemia is a common consequence of D2 receptor blockade in the tuberoinfundibular dopaminergic pathway [1], [2] by antipsychotic drugs. However, increased prolactin levels have also been reported in drug-naïve patients with a first psychotic episode or at risk mental states [3]–[5]. The mechanisms that mediate the increase of prolactin levels in psychotic subjects not receiving antipsychotic drugs are poorly understood. Moreover, prolactin levels may be increased by stress [6], which may in turn contribute to the increased prolactin levels in drug-naïve psychotic populations.

The most studied consequences of hyperprolactinaemia in psychotic subjects are amenorrhoea, galactorrhoea, sexual impairment and infertility [7], [8]. A recent study conducted in non-psychiatric population suggests that increased prolactin may have negative effects on cognition [9]. This prospective study examined the cognitive changes during late pregnancy and the early postpartum period, and their possible association with fluctuating hormone levels (estradiol, progesterone, testosterone, prolactin and cortisol). A total of 55 pregnant women and 21 controls were studied, with a neuropsychological assessment during the third trimester of pregnancy and retest during the early postpartum period. They concluded that very high and very low levels of cortisol were associated with poorer performance in certain cognitive domains, but the most novel finding was that they found a negative linear association between prolactin levels and executive function scores, suggesting that higher levels of prolactin are detrimental to executive function abilities. Animal studies also support a role for prolactin in the modulation of non-spatial cognitive tasks [10]. In this recent study, the induction of hyperprolactinaemia in male rats receiving pituitary grafts was associated with impaired object recognition. Other studies have reported an association between low gonadal steroid levels and poorer cognitive abilities [11], [12]. To our knowledge, there are no studies addressing whether high prolactin levels often found in psychotic patients can contribute to the cognitive impairment of patients with a psychotic disorder.

Subjects with schizophrenia show mild to moderate cognitive impairment, and perform an average of 1.5 to 2 standard deviations below population norms [13], [14]. These cognitive alterations appear before the onset of the first psychotic episode [15], [16] and are an important determinant of functional outcome [17]. Early intervention in psychosis is a novel approach to mental health care that includes treatment of both psychotic disorders at first years after the onset (defined as a critical period in the first 3 to 5 years after the onset) as well as subjects with prodromal symptoms who are at risk for psychosis (high-risk, HR). Early intervention services have been developed to reduce the duration of untreated psychosis, a variable that has been associated with a poorer prognosis of the illness and poorer cognitive performance [18].

Some studies have shown that atypical antipsychotic drugs have a better cognitive profile than typicals [19]–[22], but this remains controversial. Antipsychotic drugs differ in their affinity at muscarinic receptors, with detrimental effects on cognitive abilities in those antipsychotics with higher anticholinergic activity [23]. However, whether different antipsychotic medications exert any benefits on cognitive performance remains questionable [24], [25]. Based on the degree of blockade of D2 receptors at this pathway, the risk of hyperprolactinaemia differs among different antipsychotics, being greater for typical antipsychotics and some atypicals including risperidone and paliperidone [26], [27]. Also, concomitant treatment with anticholinergics or benzodiazepines can also have deleterious effects on cognition [28]–[30].

The main aim of our study was to explore whether prolactin levels are associated with poorer cognitive functioning in subjects with early psychoses, including both first episode of psychosis and high-risk subjects. We hypothesized that increased prolactin is associated with poorer cognitive performance in subjects with early psychoses. We also aimed to determine whether prolactin may mediate the relationship between antipsychotic drugs and cognition while adjusting for other potential contributors (e.g. adjunctive treatment with benzodiazepines or anticholinergic drugs).

## Materials and Methods

### Ethics Statement

All procedures are in accordance with the Declaration of Helsinki. Ethical approval was obtained from the Committee for Ethical Clinical Investigation of the *Hospital Sant Joan de Reus*. The capacity of the patients to provide informed consent was evaluated and confirmed by a psychiatrist. After complete description of the study to the subjects, written informed consent was obtained from all participants (or their guardians if the patients had a compromised capacity). All potential participants who declined to participate or otherwise did not participate were not disadvantaged in any way by not participating in the study.

### Participants

We studied 78 outpatients with an early psychosis, aged between 18 and 35 years old, attending the Early Psychosis Program from Reus (HPU Institut Pere Mata, Spain). We included a control group of 29 healthy subjects (HS) that were recruited among patients’ friends and non-genetic relatives, and screened to rule out past or current history of psychiatric disorder.

Early psychosis patients were divided into two different clinical populations: 1) High risk for psychosis (HR, subjects with prodromal psychotic symptoms fulfilling set criteria for At Risk Mental State [31], N = 23); 2) Psychotic Disorder with less than 3 years from the onset of the illness (PD, N = 55). DSM-IV diagnoses for the PD group were: schizophreniform disorder (N = 11), schizophrenia (N = 10), schizoaffective disorder (N = 3), psychotic disorder not otherwise specified (N = 31). Exclusion criteria were: pregnancy, mental retardation, severe head injury or neurological disease, active glucocorticoid treatment, language difficulties, visual impairment and alcohol, cocaine or heroin dependence.

### Characteristics of Patients

All subjects were assessed with the Schedules for Clinical Assessment in Neuropsychiatry [32]. OPCRIT checklist v.4.0. (available at http://sgdp.iop.kcl.ac.uk/opcrit/) was used to generate DSM-IV diagnoses for psychotic disorders. HR subjects were also assessed with the Comprehensive Assessment of At Risk Mental States, to ensure that subjects met criteria for any of the three HR groups defined by At Risk Mental State criteria [31].

Socio-demographic and clinical variables related to psychosis (age at onset, antipsychotic treatment, substance use) were assessed by semistructured interview. Tobacco, cannabis and alcohol consumption were registered in cigarettes/day, joints/day and standard units/day respectively. Positive and Negative Symptom Scale [33] was administered to explore positive, negative and overall psychotic symptoms. Calgary Depression Scale [34] was administered to explore depressive symptoms. These scales were administered the same day of the cognitive assessment.

Psychopharmacological treatment at neuropsychological assessment was registered. Of all 78 patients, 27 (34.6%) were not taking antipsychotics, 42 (53.8%) were on antipsychotic monotherapy (risperidone [n = 20], paliperidone [n = 5], olanzapine [n = 13], quetiapine [n = 1], aripiprazole [n = 8]) and 9 (11.5%) were on polytherapy.

Each antipsychotic dose was transformed into chlorpromazine equivalents in mg/day [35]. We recoded chlorpromazine equivalent doses into three different variables taking into account the mechanism of action of each antipsychotic and its effects on prolactin and anticholinergic activity: 1) risperidone/paliperidone (prolactin elevating without anticholinergic activity), 2) olanzapine/quetiapine/clozapine (prolactin sparing with anticholinergic activity) and 3) aripiprazole (prolactin sparing without anticholinergic activity). Benzodiazepine treatment was registered in diazepam equivalent doses. Biperiden dose was registered in mg/day. Antidepressant treatment was registered as fluoxetine equivalents in mg/day.

### Cognitive Assessment

The MATRICS Consensus Cognitive Battery (MCCB) was administered to explore neuropsychological functioning [36]. This cognitive battery has demonstrated practicality of administration, high test-retest reliability, small practice effects, small ceiling effects and relationship to functional outcome. The MCCB contains 10 tests to measure cognitive performance in 7 cognitive domains: speed processing, attention/vigilance, working memory, verbal learning, visual learning, reasoning and problem solving, and social cognition (Table S1). A composite Score is obtained, which combines the individual scores of the 10 tests and scores them on a normative scale to derive a T-score, where the mean is 50 and a standard deviation is 10 for the composite. Normative data for the MCCB has been obtained in Spain [37], suggesting that significant age, gender, and education effects are comparable to those effects described for the original standardized English version in the U.S. All neuropsychological assessments were performed in the morning, with starting times between 9 h and 12 h. Cognitive testing in PD was assessed when they were clinically stable.

### Hormonal Measures

A fasting blood sample was obtained in the morning between 8∶30 h and 9∶30 h in resting conditions, to determine unstimulated plasma prolactin and total cortisol levels. Participants were told to avoid stressful activities (sports, physical exercise) or breast stimulation in the 12 hours prior to blood sampling. Prolactin and cortisol concentrations were measured using the Maglumi 2000 Analyzer chemiluminescence immunoassay system (SNIBE Co, Ltd, Guandong, China). The sensitivity of the prolactin assay was 1.77 µg/L.

### Statistical Analysis

The SPSS version 19.0 software (SPSS Inc., Chicago, Illinois, USA) was used to carry out the statistical analyses. As prolactin levels, PANSS and CDS scores were skewed, we log transformed (ln) these variables to reduce skewness. Chi-square tests and one-way ANOVA were used to compare categorical and continuous data between groups. Post-hoc ANOVA analyses were adjusted with a Bonferroni correction. Spearman correlation was used to explore the association between continuous or ordinal variables. Significance was set at p<0.05 (two-tailed). For all statistical analyses we used MCCB T-scores corrected for age, gender and education level.

We first divided the sample by diagnostic group (HS, PD and HR). A stratified analysis by diagnostic group was conducted to explore the association between prolactin levels and cognitive measures in each subgroup of participants.

A multiple linear regression analysis was performed in all patients (including both PD and HR subjects) to explore the relationship between plasma prolactin levels (main independent variable) and MCCB cognitive domains (dependent variable) while controlling for covariables such as other psychopharmacological treatments, psychopathological status, smoking and other substance use (cannabis and alcohol) and cortisol.

Furthermore, those MCCB domains that showed a significant association with prolactin in the multivariate analyses were also tested with a mediation analysis to explore whether the effect of antipsychotic treatment on cognition could be mediated by prolactin levels and benzodiazepine treatment. We conducted a mediation analysis according to Baron and Kenny [38] and used bootstrapping to test the indirect effect of mediation [39]. A description of mediation analysis is shown in Box S1.

We used a SPSS macro [40] that allows the inclusion of multiple mediators and covariates. In this mediation analysis, we decided to include risperidone/paliperidone dose as the main independent variable because its elevating effect on prolactin levels. MCCB cognitive T-score (e.g. Speed of Processing) was used as the dependent variable. Two potential mediators were considered: prolactin and benzodiazepine treatment. We included as covariates other antipsychotic drugs, biperiden and antidepressant treatments. Significance of the indirect effects in this model was tested by bootstrapping.

R and ggplot2 package (http://www.r-project.org/) were used to draw scatterplot figures exploring the association between prolactin and MCCB cognitive domains.

## Results

### Univariate Analyses

Clinical characteristics of the sample by diagnostic group are described in Table 1. As expected, we found that HS performed significantly better than PD in all cognitive domains, and better than HR in most domains (Table 1). There were no significant differences in psychopathological scales between PD and HR. PD subjects were more frequently treated with antipsychotics, when compared to HR. However, the latter group was more frequently treated with antidepressants.

**Table 1:** Clinical and cognitive variables by diagnostic groups.

	Healthy subjects (HS)N = 29	High-Risk (HR)N = 23	Psychotic disorder(PD) N = 55	P value†
Age (years)	26.4 (4.3)	22.5 (4.3)	24.5 (5.3)	0.019^b^
Female gender	14 (48.3)	5 (21.7)	25 (45.5)	0.100
PANSS positive	–	9.9 (2.8)	10.3 (3.6)	0.094
PANSS negative	–	13.2 (6.2)	14.4 (5.5)	0.870
PANSS general	–	28 (7.4)	25.6 (7.4)	0.180
Calgary Depression Scale	–	3.8 (0.8)	3.3 (0.4)	0.555
Plasma prolactin (µg/L)	15.5 (6.3)	27.5 (29.0)	44.4 (40.7)	<0.001^a^
*Plasma total cortisol (µg/dL)*	17.7 (6.1)	19.8 (1.5)	18.8 (5.5)	0.468
*MCCB cognitive domains (T-scores)*				
Speed of processing	50.8 (9.9)	41.4 (11.4)	34.5 (12.6)	<0.001^a,b^
Attention and vigilance	44.9 (9.4)	37.0 (10.1)	36.8 (9.5)	0.001^a,b^
Working memory	44.6 (9.6)	37.0 (8.2)	36.7 (9.1)	0.002^a,b^
Verbal learning	48.9 (9.8)	43.9 (7.7)	41.9 (7.9)	<0.001^a^
Visual learning	50.5 (6.2)	44.9 (9.3)	37.3 (12.3)	<0.001^a,b^
Reasoning and problem solving	49.0 (8.5)	44.5 (10.2)	41.8 (9.7)	0.006^a^
Social cognition	52.1 (10.6)	47.4 (11.4)	39.5 (11.1)	<0.001^a,b^
Composite factor (global)	47.6 (8.6)	37.3 (7.5)	32.0 (9.4)	<0.001^a,b^
*Psychopharmacological treatment* ‡				
Antipsychotic treatment				
None	29 (100)	17 (73.9)	10 (18.2)	<0.001
Risperidone/Paliperidone in monotherapy	0 (0)	3 (13.0)	17 (30.9)	
Olanzapine/Quetiapine in monotherapy	0(0)	0 (0)	14 (25.5)	
Aripiprazole in monotherapy	0 (0)	2 (8.7)	6 (10.9)	
Polytherapy	0 (0)	1 (4.3)	8 (14.5)	
Benzodiazepine treatment	0 (0)	3 (13.0)	12 (21.8)	0.370
Biperiden treatment	0 (0)	1 (4.3)	7 (12.7)	0.266
Antidepressant treatment	0 (0)	14 (60.9)	8 (14.5)	<0.001
*Substance use*				
Tobacco (cigarettes/day)	1.8 (4.6)	3.3 (6.1)	9.4 (9.9)	<0.001^a,c^
Cannabis (joint/day)	0 (0)	0.4 (1.5)	1.9 (4.6)	0.035
Alcohol (standard units/day)	0.1 (0.4)	0 (0)	0.8 (2.4)	0.097

Data are mean (SD) or N (%). Four participants (3 PD, 1 HR) had missing data for prolactin analyses.

Abbreviation: PANSS = Positive and Negative Syndrome Scale; MCCB = MATRICS Consensus Cognitive Battery.

† One-way ANOVA was used to compare continuous data among groups. Chi-square test was used to compare categorical data among groups.

‡ Psychopharmacologic treatment was compared between PD and HR groups (HS were excluded in the comparison).

Significant ANOVA post-hoc analyses (with Bonferroni adjustment) are highlighted: ^a^ HS vs PD, ^b^HS vs HR, ^c^PD vs HR.

**Figure 1:**
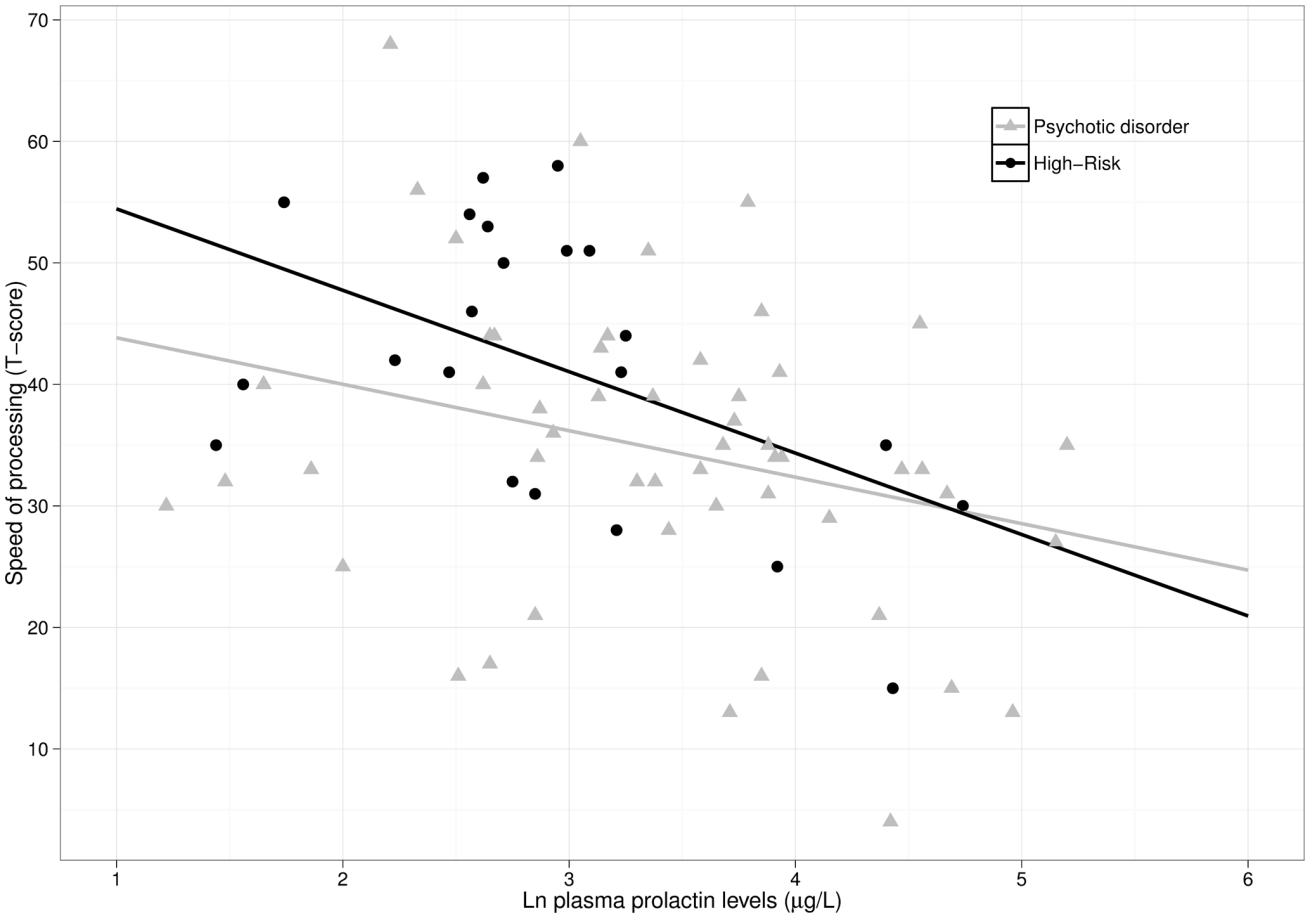
Scatter plot of the relationship between prolactin levels and speed of processing T-scores between diagnostic groups (psychotic disorder vs high-risk).

In the stratified analysis by diagnostic group, prolactin levels were significantly associated with processing speed in both PD and HR subjects (Table 2). In HR subjects, prolactin levels were also related with poorer cognitive performance in reasoning and problem solving, and global cognition. A scatter plot by diagnostic group (PD vs HR) is shown in Figure 1. We also performed another analysis including both HR and PD together (Table S2). In this analysis, prolactin was positively associated with risperidone/paliperidone and benzodiazepine doses and negatively associated with speed of processing.

**Table 2:** Correlation between prolactin levels and MCCB cognitive domains (T-scores) and psychopharmacological treatments.

	Healthy subjects (N = 29) Prolactin*		High-Risk (N = 23) Prolactin*		Psychotic disorder (N = 55) Prolactin*	
	r	P	r	P	r	P
Speed of processing	−0.023	0.906	−0.498	0.018	−0.286	0.040
Attention and vigilance	0.040	0.838	−0.128	0.569	−0.070	0.625
Working memory	−0.043	0.826	−0.150	0.504	0.119	0.400
Verbal learning	−0.302	0.111	−0.236	0.290	−0.037	0.795
Visual learning	−0.159	0.411	−0.303	0.181	0.016	0.910
Reasoning and problem solving	0.083	0.670	−0.449	0.036	0.001	0.992
Social cognition	0.191	0.321	0.212	0.370	0.119	0.420
Composite (global)	−0.042	0.830	−0.458	0.049	0.137	0.369
Risperidone/Paliperidone dose†			0.372	0.088	0.479	<0.001
Olanzapine/Clozapine/Quetiapine dose†			0.074	0.744	0.131	0.355
Aripiprazole dose†			−0.263	0.237	−0.203	0.149
Benzodiazepine dose‡			−0.049	0.828	0.270	0.053
Biperiden dose (mg/day)			0.389	0.074	0.122	0.389
Antidepressant dose§			0.135	0.549	−0.030	0.831

Stratified analysis by diagnostic group.

^*^ Log transformed (ln) values of prolactin.

† In equivalents of chlorpromazine (mg/day).

‡ In equivalents of diazepam (mg/day).

§ In equivalents of fluoxetine (mg/day).

### Multivariate Analyses

In the multivariate analysis conducted in both HR and PD groups, the significant negative association between prolactin and speed of processing was maintained after adjusting for psychopharmacological treatments, smoking and substance use (cannabis and alcohol consumption), severity of psychotic symptoms and cortisol levels (Table 3). As shown in this table, both prolactin and risperidone/paliperidone treatment were related to a poorer processing speed (Model 2). However, when other drugs were included in the equation (Model 3), impaired processing speed was associated with benzodiazepine treatment but not antipsychotic doses. Other MCCB cognitive domains were not associated with prolactin levels in the multiple linear regression analyses (Table S3).

**Table 3:** Results of the multiple linear regression analysis exploring the relationship between prolactin levels and speed of processing in subjects with early psychoses.

	Model 1 unadjusted		Model 2+ antipsychotics		Model 3+ other treatments		Model 4+ psychotic symptoms, substance use and cortisol	
R^2^ of each model	0.140		0.214		0.300		0.434	
	β	P	β	P	β	P	β	P
Prolactin (ln)	−0.374	0.001	−0.256	0.044	−0.245	0.046	−0.283	0.022
Risperidone/Paliperidone dose*			−0.243	0.042	−0.139	0.311	−0.003	0.986
Olanzapine/Quetiapine/Clozapine dose*			−0.140	0.210	0.037	0.769	0.131	0.297
Aripiprazole dose*			−0.152	0.176	−0.146	0.168	−0.072	0.497
Benzodiazepine treatment‡					−0.353	0.006	−0.324	0.015
Biperiden (mg/day)					−0.045	0.699	−0.034	0.773
Antidepressant treatment†					0.066	0.547	0.067	0.537
PANSS - positive subscore (ln)							−0.192	0.143
PANSS – negative subscore (ln)							−0.283	0.035
PANSS – general subscore (ln)							0.103	0.434
Tobacco (cigarettes/day)							−0.129	0.316
Cannabis (joints/day)							0.060	0.610
Alcohol (standard units/day)							0.042	0.727
Cortisol (µg/dL)							0.029	0.794

T-score (adjusted for age, gender and education level) in the speed of processing MCCB domain was considered the dependent variable.

Abbreviations: PANSS = Positive and Negative Syndrome Scale; *β = *Standardized beta coefficient; MCCB = MATRICS Consensus Cognitive Battery.

* In chlorpromazine equivalents, mg/day.

‡ In diazepam equivalents, mg/day.

† In fluoxetine equivalents, mg/day.

**Figure 2:**
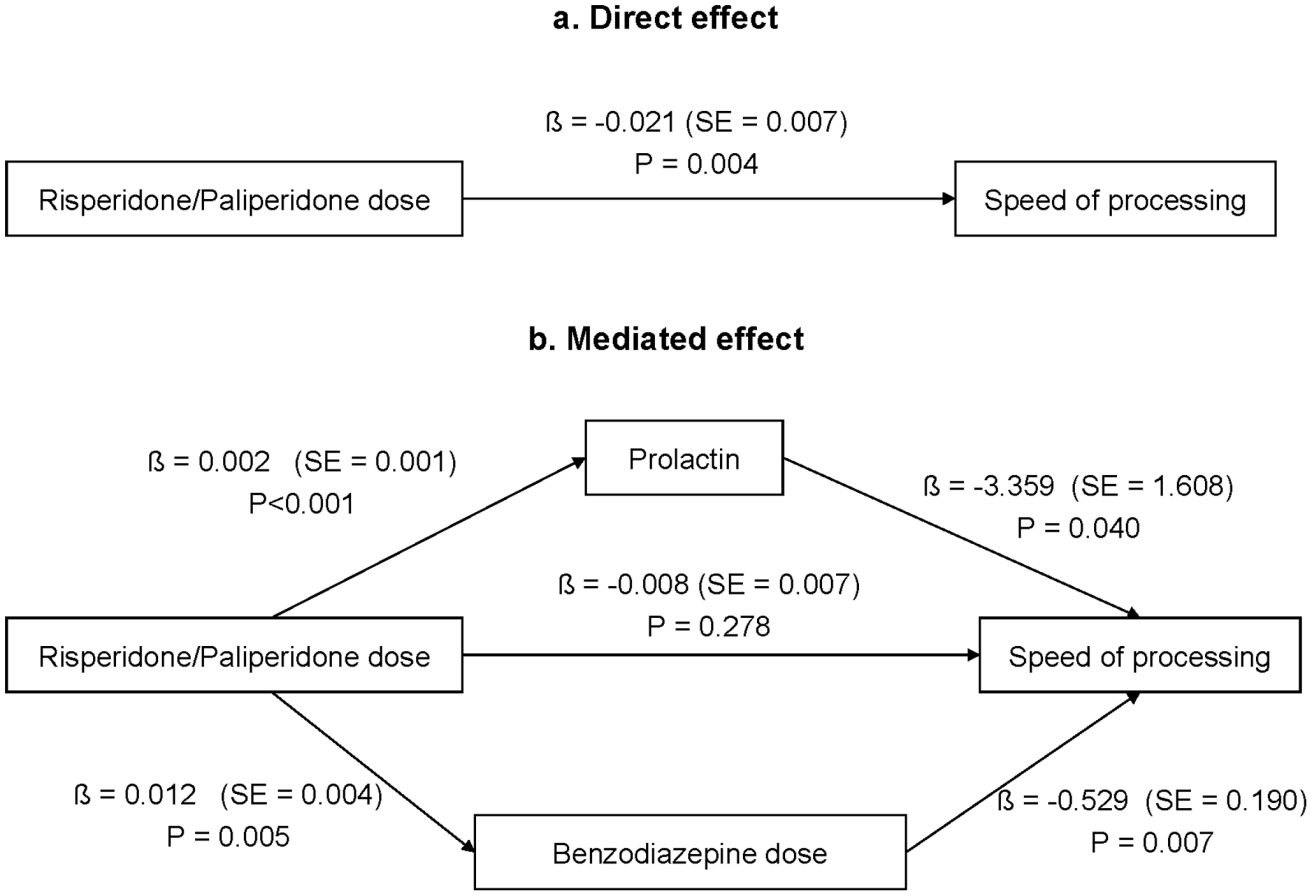
Results of the mediation analysis exploring the relationship between risperidone/paliperidone dose and processing speed in subjects with early psychoses. Log transformed (ln) levels of prolactin were used in the mediation analysis. The mediated effect (b) was adjusted for the following covariates: olanzapine/clozapine/quetiapine dose (β = 0.002, SE = 0.008, P = 0.848), aripiprazole dose (β = −0.016, SE = 0.011, P = 0.151), biperiden dose (β = −0.567, SE = 1.486, P = 0.704) and antidepressant dose (β = 0.048, SE = 0.101, P = 0.638). Abbreviations: β = unstandardized regression coefficient; SE = standard error.

In the mediation analysis, we tested two potential mediators (prolactin and benzodiazepine treatment) in the relationship between risperidone/paliperidone doses and processing speed (Figure 2). In the unadjusted model (a), risperidone/paliperidone treatment was negatively associated with speed of processing. This effect was fully mediated by both prolactin and benzodiazepine treatment (b), as the relationship between antipsychotic treatment and speed of processing lost its significance when these two mediators were included in the equation. In this mediated analysis, other antipsychotics, antidepressants and biperiden were included as covariates, thus the results are adjusted for these psychopharmacological drugs. As a multiple mediation model is analogous to conducting a regression analysis with several predictors testing the total indirect effect of the independent variable (risperidone/paliperidone treatment) on the dependent variable (speed of processing), both prolactin and benzodiazepines are independently associated with speed of processing. The indirect effects of both variables account for all the observed relationship between risperidone/paliperidone treatment and speed of processing. Bootstrap results for indirect effects were significant for both prolactin (95% CI: −0.165 to –0.001) and benzodiazepine treatment (95% CI: −0.167 to –0.002).

## Discussion

Our study suggests that increased prolactin levels are associated with impaired processing speed independent of antipsychotic drugs in subjects with early psychosis. In HR subjects only, increased prolactin was also associated with impaired reasoning and problem solving and poorer general cognition. The results of the mediation analysis also showed that the effect of risperidone/paliperidone treatment on speed of processing is mediated by both prolactin levels and benzodiazepine treatment. To our knowledge, this is the first study to highlight prolactin as an important contributor to cognitive impairment in subjects with psychosis.

Regarding speed of processing, our results are consistent with previous studies reporting that processing speed is the first cognitive domain to be affected in psychotic disorders at early stages [41] and in at risk mental states subjects [42]. This cognitive domain, that may be considered a core feature of schizophrenia neurocognitive impairment, is thought to mediate the relationship between cognitive symptoms and functional outcome in schizophrenia [43]. In contrast to other cognitive domains, speed of processing is considered a “system based” domain, reflecting a process of integration and coordination between distributed brain networks [44]. This is in line with the disconnection hypothesis of schizophrenia, which asserts that impaired communication within the brains of schizophrenia patients occurs when there is focal disruption that adversely affects the entire network. Speed of processing deficits may point to aberrant functional connectivity within and between whole brain neural systems, rather than indexing impairment in discrete neural networks [45].

Although antipsychotic drugs are a common cause of hyperprolactinaemia, other studies in drug-naïve patients have also shown increased prolactin levels in PD and HR subjects [3]–[5]. Interestingly, in our study the relationship between prolactin and processing speed was also found in HR subjects, most of whom were not receiving antipsychotic treatment. Moreover, in the multivariate analyses, the association between prolactin levels and impaired processing speed remained significant after adjusting for psychopharmacological treatments. Prolactin is a hormone that may be raised in stressful situations. We accounted for this by controlling for cortisol levels in the multivariate analysis, and the effect of prolactin on cognition was independent of hypothalamic-pituitary-adrenal axis activity.

Antipsychotic-induced hyperprolactinaemia, which is caused by tuberoinfundibular blockade of D2 receptors, may be reflecting the blockade of D2 receptors in other dopaminergic pathways including the striatum, which causes extrapyramidal symptoms, or the mesocortical pathway, that may be related to worsening in cognitive and negative symptoms. The mediated analysis suggests that the negative effect of risperidone/paliperidone on processing speed is mediated by both prolactin and benzodiazepines. This could be explained by the prolactin-elevating profile of these antipsychotics as well as the induction of extrapyramidal symptoms (e.g. akathysia) that are often treated with benzodiazepines.

Our study suggests that prolactin may be considered as a biomarker that is associated with impaired processing speed in subjects with early psychoses. This finding may have important clinical implications. Further studies are needed to explore whether a reduction in prolactin levels by optimizing psychopharmacological treatment leads to an improvement in processing speed.

The main limitation of our study is the cross-sectional design that does not allow us to infer causality. Further prospective studies may overcome this limitation by repeatedly assessing prolactin levels and cognitive performance over time to explore whether persistent hyperprolactinaemia is a risk factor for cognitive decline in subjects with psychoses. We did not control for other hormones that may affect cognition such as sex steroids. Hyperprolactinaemia inhibits the hypothalamic-pituitary-gonadal axis [7], and hypogonadism secondary to hyperprolactinaemia may contribute to the negative effects of prolactin on cognition. However, in order to control for these variables, larger samples are required because a sex-stratified analysis is necessary (controlling for estradiol in women and testosterone in men).

We designed a pragmatic study with consecutive sampling in an Early Intervention Service. For this reason, the sample of our study included both HR subjects and psychotic disorders at early stages, and patients were treated with different antipsychotics that were chosen by clinicians based on the clinical routine practice. HR subjects and PD patients exhibited similar PANSS scores. As our Early Intervention Service is an outpatient service, some patients who have been previously admitted to the referral Acute Psychiatric Unit are stabilized before attending our service. Moreover, patients need to be informed of the research project and must sign the informed consent; thus in most cases, PD patients are clinically stable at recruitment. As most PD patients have been treated before entering the study, the PANSS scores reflect the psychopathological state at the neuropsychological assessment (not at the acute phase of the illness). These characteristics could explain why both groups (HR and PD) exhibited similar PANSS scores. Although HR group shares some characteristics with PD group at early stages such as cognitive impairment, only 30% develop psychosis at one year [46]. From our cross-sectional design we do not know whether prolactin could be a biomarker related to the risk of transition to psychosis. Future prospective studies are needed to clarify this question. Finally, the sample of our study was composed by outpatients, and none of whom were receiving first-generation antipsychotics. Thus, our results may not be generalizable to other populations including inpatients, psychotic patients with a longer duration of illness or patients taking typical antipsychotics. In fact, a recent study conducted in patients with chronic schizophrenia showed that switching to aripiprazole led to a decrease in prolactin levels but was not associated with cognitive improvement [47]. Our results should be replicated in other samples to draw definite conclusions.

However, several strengths of our study need to be highlighted: 1) our study is the first to describe the potential role of prolactin on cognition in subjects with psychoses, 2) we have used a standardized neurocognitive battery (MCCB) to assess cognition, 3) the sample included subjects with a short duration of the illness, and 4) we controlled for potential confounders such as concomitant antipsychotic medication (chlorpromazine equivalents) by differentiating antipsychotics depending upon their mechanism of action, as well as controlling for benzodiazepines that have been suggested as moderating factors of processing speed [48], and 5) we also controlled for smoking status, which has been describe to modify prolactin levels [49] and to interfere with cognition in early psychosis patients [50].

## Conclusions

In summary, our study suggests that increased prolactin levels are associated with impaired processing speed in early psychosis and that also mediate the negative effects of prolactin elevating antipsychotics on processing speed. If these results are replicated in further studies, hyperprolactinaemia may be considered as a potential contributor to cognitive deterioration in psychotic subjects and strategies targeting reduction of prolactin levels may improve cognition in this population.

## Supporting Information

Table S1 MATRICS Consensus Cognitive Battery tests and cognitive domains.
(DOC)

Table S2 Correlations between prolactin levels, psychopharmacological treatment, psychopathological status and MCCB cognitive domains in subjects with early psychosis.
(DOC)

Table S3 Multiple regression analyses exploring the relationship between prolactin levels in plasma and MCCB Cognitive domains in subjects with early psychosis.
(DOC)

Box S1 Explanation of the mediation analysis.
(DOC)
