# Supplementary material for: Increased Prolactin Levels Are Associated with Impaired Processing Speed in Subjects with Early Psychosis
Source: PLoS One. 2014 Feb 24;9(2):e89428. doi: 10.1371/journal.pone.0089428 (PMC3933530; doi:10.1371/journal.pone.0089428)
Supplement: Table S1 — MATRICS Consensus Cognitive Battery tests and cognitive domains. (DOC) [file pone.0089428.s001.doc]

Table S1. MATRICS Consensus Cognitive Battery tests and cognitive domains.

| Cognitive domain | Neuropsychological test |
| --- | --- |
| Speed of processing | Brief Assessment of Cognition in Schizophrenia-Symbol Coding |
|  | Category Fluency-Animal naming |
|  | Trail Making Test Part A |
| Attention and vigilance | Continuous Performance Test-Identical Pairs |
| Working memory | WMS-III Spatial Span |
|  | University of Maryland Letter-Number Span |
| Verbal learning | Hopkins Verbal Learning Test-Revised |
| Visual learning | Brief Visuospatial Memory Test-Revised |
| Reasoning and problem solving | Neuropsychological Assessment Battery-Mazes |
| Social cognition | Mayer-Salovey-Caruso Emotional Intelligence Test-Managing Emotions |
