# Supplementary material for: Increased Prolactin Levels Are Associated with Impaired Processing Speed in Subjects with Early Psychosis
Source: PLoS One. 2014 Feb 24;9(2):e89428. doi: 10.1371/journal.pone.0089428 (PMC3933530; doi:10.1371/journal.pone.0089428)
Supplement: Table S2 — Correlations between prolactin levels, psychopharmacological treatment, psychopathological status and MCCB cognitive domains in subjects with early psychosis. (DOC) [file pone.0089428.s002.doc]

|  | Prolactin | Risperidone/Paliperidone dose**^†^** | Olanzapine/Clozapine/ Quetiapine dose**^†^** | Aripiprazole dose**^†^** | Benzodiazepine dose^‡^ | Biperiden dose (mg/day) | PANSS-P | PANSS-N | PANSS-G | CDS |
| --- | --- | --- | --- | --- | --- | --- | --- | --- | --- | --- |
| Prolactin | - | 0.641*** | 0.253* | -0.131 | 0.286** | 0.264** | 0.130 | 0.137 | 0.136 | 0.140 |
| Risperidone/Paliperidone dose**^†^** | 0.641*** | - | 0.036 | -0.132 | 0.291** | 0.368*** | 0.090 | 0.155 | 0.048 | 0.092 |
| Olanzapine/Clozapine/Quetiapine dose**^†^** | 0.253* | 0.036 | - | -0.041 | 0.308** | -0.059 | -0.233* | 0.218 | 0.184 | -0.064 |
| Aripiprazole dose**^†^** | -0.131 | -0.132 | -0.041 | - | -0.053 | 0.019 | 0.009 | 0.055 | -0.052 | -0.046 |
| Benzodiazepine dose^‡^ | 0.286** | 0.291** | 0.308** | -0.053 | - | -0.007 | 0.239* | 0.115 | 0.271* | 0.036 |
| Biperiden dose (mg/day) | 0.264** | 0.368*** | -0.059 | 0.019 | -0.007 | - | 0.004 | 0.044 | -0.045 | 0.181 |
| Speed of Processing | -0.423*** | -0.477*** | -0.216* | -0.167 | -0.255** | -0.207* | -0.283* | -0.405*** | -0.193 | -0.091 |
| Attention and vigilance | -0.196* | -0.397*** | 0.007 | -0.122 | -0.185 | -0.059 | -0.230* | -0.363** | -0.299** | -0.219 |
| Working memory | -0.078 | -0.127 | -0.242* | 0.019 | -0.118 | 0.028 | -0.070 | 0.266* | -0.141 | 0.103 |
| Verbal learning | -0.210* | -0.243* | -0.258** | -0.141 | -0.187 | 0.017 | -0.178 | -0.203 | -0.159 | -0.142 |
| Visual learning | -0.335** | -0.312** | -0.199* | 0.241* | -0.256** | -0.085 | -0.127 | -0.366** | -0.204 | -0.144 |
| Reasoning and problem solving | -0.174 | -0.232* | -0.028 | -0.050 | -0.173 | -0.099 | 0.119 | -0.075 | 0.037 | 0.044 |
| Social cognition | -0.029 | -0.255* | -0.173 | 0.060 | -0.106 | -0.111 | -0.208 | -0.368** | -0.103 | -0.022 |
| Composite score | -0.254* | -0.395*** | -0.116 | -0.151 | -0.168 | -0.202* | -0.229 | -0.429*** | -0.220 | -0.198 |

Table S2. Correlations between prolactin levels, psychopharmacological treatment, psychopathological status and MCCB cognitive domains in subjects with early psychosis.

*p<0.05; ** p<0.01; *** p<0.001

^†^ In equivalents of chlorpromazine (mg/day)

^‡^ In equivalents of diazepam (mg/day)

Abbreviations: MCCB= Matrics Consensus Cognitive Battery; PANSS= Positive and Negative Syndrome Scale (PANSS-P: Positive subscore; PANSS-N: Negative subscore; PANSS-G: General subscore); CDS= Calgary Depression Score
