# Supplementary material for: Increased Prolactin Levels Are Associated with Impaired Processing Speed in Subjects with Early Psychosis
Source: PLoS One. 2014 Feb 24;9(2):e89428. doi: 10.1371/journal.pone.0089428 (PMC3933530; doi:10.1371/journal.pone.0089428)
Supplement: Table S3 — Multiple regression analyses exploring the relationship between prolactin levels in plasma and MCCB Cognitive domains in subjects with early psychosis. (DOC) [file pone.0089428.s003.doc]

Table S3. Multiple regression analyses exploring the relationship between prolactin levels in plasma and MCCB Cognitive domains in subjects with early psychosis.

|  | **Model 1 unadjusted** | | **Model 2 + antipsychotic treatment*** | | **Model 3 + other treatments^†^** | | **Model 4 + severity of psychotic symptoms, substance use and cortisol**^‡^ | |
| --- | --- | --- | --- | --- | --- | --- | --- | --- |
|  | *β* | *p-value* | *β* | *p-value* | *β* | *p-value* | *β* | *p-value* |
| **Speed of processing** | -0.374 | 0.001 | -0.256 | 0.044 | -0.245 | 0.046 | -0.283 | 0.022 |
| **Attention and vigilance** | -0.110 | 0.360 | 0.061 | 0.650 | 0.071 | 0.590 | 0.115 | 0.394 |
| **Working memory** | 0.039 | 0.745 | 0.166 | 0.226 | 0.172 | 0.204 | 0.180 | 0.218 |
| **Verbal learning** | -0.113 | 0.344 | -0.044 | 0.752 | -0.042 | 0.757 | -0.001 | 0.996 |
| **Visual learning** | -0.122 | 0.312 | 0.012 | 0.925 | 0.012 | 0.929 | 0.006 | 0.963 |
| **Reasoning and problem solving** | -0.146 | 0.225 | -0.164 | 0.253 | -0.156 | 0.268 | -0.193 | 0.193 |
| **Social cognition** | 0.077 | 0.537 | 0.249 | 0.079 | 0.248 | 0.085 | 0.226 | 0.104 |
| **Composite factor (global)** | -0.058 | 0.653 | 0.003 | 0.982 | 0.017 | 0.909 | -0.002 | 0.987 |

^*^ Antipsychotic treatment was converted to equivalents of chlorpromazine (in mg per day)

† Benzodiazepine (in equivalents of diazepam, mg per day), biperiden (mg per day) and antidepressant (in equivalents of fluoxetine, mg per day) treatments were included as covariates in this step.

‡ PANSS positive, negative and general subscores, substance use (tobacco, cannabis and alcohol) and plasma total cortisol were included as covariates in this step.

Abbreviations:

MCCB= Matrics Consensus Cognitive Battery; *β=* Standardized beta coefficient.
