# Supplementary material for: Increased Prolactin Levels Are Associated with Impaired Processing Speed in Subjects with Early Psychosis
Source: PLoS One. 2014 Feb 24;9(2):e89428. doi: 10.1371/journal.pone.0089428 (PMC3933530; doi:10.1371/journal.pone.0089428)
Supplement: Box S1 — Explanation of the mediation analysis. (DOC) [file pone.0089428.s004.doc]

Box S1. Explanation of the mediation analysis.


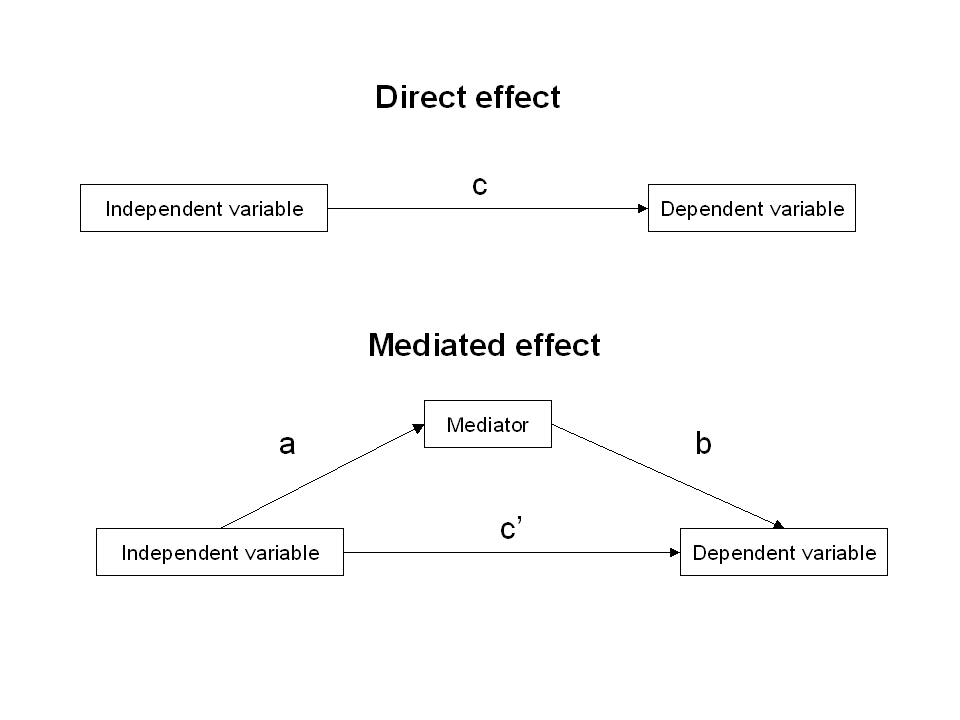


According to Baron and Kenny (Baron & Kenny, 1986), the following regression equations should be estimated in a mediation analysis:

1. the association between the independent variable and the dependent variable (c)
2. the association between the independent variable and the mediator (a)
3. the association between the mediator and the dependent variable (b), controlling for the effect of the independent variable (c’).

In order that mediation exists, the strength of the relation between the independent variable and the dependent variable (c) is significantly reduced when the mediator is added to the model (c’). If perfect mediation exists, the effect of the independent variable on the dependent variable controlling for the mediator should be zero.

The amount of mediation is called indirect effect. An increasingly popular method of testing the indirect effect is bootstrapping (Shrout & Bolger, 2002). Bootstrapping is a non-parametric method based on resampling with replacement which is done many times. From each of these samples the indirect effect is computed and a sampling distribution can be empirically generated. Because the mean of the bootstrapped distribution will not exactly equal the indirect effect a correction for bias is usually made. With the distribution, a confidence interval (CI), a p value, or a standard error can be determined. Very typically a CI is computed and it is checked to determine if zero is in the interval. If zero is not in the interval, then the researcher can be confident that the indirect effect is different from zero (For a full explanation of the mediation analysis, see http://davidakenny.net/cm/mediate.htm).

References:

Baron, R.M., Kenny, D.A., 1986. The moderator-mediator variable distinction in social psychological research: conceptual, strategic, and statistical considerations. J.Pers.Soc.Psychol. 51 (6) 1173-1182.

Shrout, P.E., Bolger, N., 2002. Mediation in experimental and nonexperimental studies: new procedures and recommendations. Psychol.Methods. 7 (4) 422-445.
